# Epidemiology of Pediatric Traumatic Brain Injury at Sylvanus Olympio University Hospital of Lomé in Togo

**DOI:** 10.1155/2019/4038319

**Published:** 2019-08-01

**Authors:** Pilakimwe Egbohou, Tabana Mouzou, Pikabalo Tchetike, Hamza Doles Sama, Sarakawabalo Assenouwe, Gnimdou Akala-Yoba, Lonlongnon Randolph, Kadjika Tomta

**Affiliations:** Anesthesiology and Intensive Care Department of Sylvanus Olympio University Hospital, University of Lome, Lome, Togo

## Abstract

**Introduction:**

Severe pediatric traumatic brain injury (pTBI) is a leading cause of disability and death in children worldwide. Children victims of pTBI are admitted to the Sylvanus Olympio University Hospital (SOUH) at the multipurpose Intensive Care Unit (ICU). We aimed in this study to describe the epidemiologic characteristics and outcomes of pTBI patients admitted in this ICU.

**Patients and Methods:**

This study was conducted at the ICU of SOUH of Lome. It was a retrospective study based on patients' records from 0 to 15 years old admitted during the period from 1 January 2012 to 30 June 2018 (5 years and 6 months).

**Results:**

We recorded 91 pTBI included in the study. The mean age was 7.7 ± 4.3 years. The male predominated with 67.0%. Road traffic accidents were the most common cause (79.1%), followed by falls (19.8%). The average pediatric Glasgow Coma Scale (pGCS) was 6.6 ± 1.4, with a mean Injury Severity Score (ISS) of 23.1 ± 8.4. The most common brain injuries found in the CT scan were brain edema (72.9%), skull fracture (69.5%), and brain contusion (55.9%). The average duration under mechanical ventilation was 2.1 ± 2.9 days, and the mean ICU stay was 4.9 ± 4.4 days. Overall mortality was 31.9% (29 cases). Factors significantly associated (*p* < 0.05) with death were hypotension (51.7%), anemia (43.1%), hyperthermia (46.7%), GCS < 6 (64%), and ISS > 20 (48.9%).

**Conclusion:**

pTBI mortality remains high in SOUH ICU. Factors associated with mortality were secondary systemic insults, worse GCS < 6, and ISS > 20.

## 1. Introduction

Severe pediatric traumatic brain injury (pTBI) is a major public health problem and a leading cause of death and disability [1–4]. pTBI is in the foreground and presents in 80 to 90% of the severe traumas of the child and their management alone determines vital and functional prognosis [4]. They are the leading cause of child mortality and are thought to be responsible for more than half of child injury deaths [5]. Studies carried out in Africa reveal high mortality rates by severe pTBI, particularly in Senegal where the mortality rate reaches 34.8% [6]. The most common causes of unintentional head trauma in children remain road traffic accidents (RTA) and falls [6–8]. In Lome (Togo), there are neither trauma centers nor pediatric intensive care units and children with severe TBI are admitted to the multipurpose Intensive Care Unit (ICU) of the Sylvanus Olympio University Hospital (SOUH). We therefore aimed in this study to describe the epidemiological characteristics of those children with severe pTBI in this ICU and to look for factors associated with poor clinical outcomes.

## 2. Materials and Methods

This study was conducted at the multipurpose ICU of SOUH. It is an all-age 12-bedded ICU with 5 intensive care physicians and 16 nurses, equipped with a mechanical ventilator. Available monitoring in this ICU included noninvasive blood pressure, pulse oximetry, and continuous ECG recording. Neuromonitoring such as intracranial pressure monitoring or transcranial Doppler were not available. Neuroimaging tests such as CT scan were not always available at the SOUH, and patients were often transported in poor conditions to other hospitals to realize diagnostic imaging.

This study concerned the period from 1 January 2012 to 30 June 2018 (5 years and 6 months). Records of patients admitted for severe pTBI, aged 0 to 15 years old, were included. The severe pTBI was defined as traumatic injuries to the scalp, skull, and brain with a pediatric Glasgow Coma Scale (pGCS) < 9 after stabilization of vital distress. A form was used to collect data. Data collected included patient demographics, cause of injury, means of transportation to the hospital, admission pGCS, Injury Severity Score (ISS), systemic secondary insults at admission, and injuries found at the cerebral CT scan when it was performed. Endotracheal intubation, duration of mechanical ventilation, and complications, length of stay in ICU, and mortality were noted. Deaths were reviewed for associated factors. Data were studied by age groups: infants and preschoolers (age 0–4 years), school-aged children (5–9 years), and adolescents (10–15 years).

### 2.1. Statistical Analysis

The data were processed using the Statistical Package for Social Science software (version 21; IBM Corp, Armonk NY, USA). The qualitative variables were expressed as frequencies and percentages, and the quantitative variables as means ± standard deviation. Qualitative variables have been compared using chi-squared test while the means of the quantitative variables were compared using the one-way analysis of variance test (ANOVA). A *p* value ≤ 0.05 was considered significant.

## 3. Results

During this study period, 998 severe TBI patients were admitted to the ICU of SOUH. Of these, 91 (9.1%) were aged 0 to 15 years and were included in the study. The average age of injured children was 7.7 ± 4.3 years. The male sex predominated with 67.0%.  Table 1 shows demographic characteristics, causes of trauma, severity of trauma, and outcomes based on children age groups. Road traffic accidents (RTA) were the most common cause of pTBI accounting for 79.1%, followed by falls (19.8%) and 1 case (1.1%) of sports accident. These causes differed among age groups. In children under 2 years, the falls predominated with 60%, followed by RTA at 40%. In the older age groups, RTA were largely at the top of the list with 87.5% in the age group of 5–9 years and 82.8% among children aged 10–15 years.

Transport for all injured children from the trauma scene to the hospital was not medicalized, and they did not benefit from any prehospital care. pTBI was not associated with any other injury in 61%. However, an association with limbs trauma has been observed in 22% (20.9% for upper limbs and 1.1% for lower limbs), as well as facial trauma in 7.7%, back spine trauma in 1.1%, thoracic contusion in 1.1%, and abdominal contusion in 1.1%. The mean score of pGCS at admission was 6.6 ± 1.4 with a median at 7. The average ISS was 23.1 ± 8.4. The mean score of GCS and ISS did not differ among different age groups (*p* > 0.05 for the GCS and ISS, see Table 1).

Cerebral CT scan could only be performed for 59 (64.8%) patients; the others did not have the means to achieve it. Injuries found on CT scan (Table 2) were dominated by brain edema (72.9%), skull fracture (69.5%), and brain contusion (55.9%); there was no significant difference (*p* > 0.05) in occurrence of injuries among age groups. The highest mortalities were observed with diffuse axonal injuries (2/3), intraventricular hemorrhage (2/3), and intracerebral hemorrhage (4/8).

Mechanical ventilation was performed under fentanyl and diazepam sedation. The mean length of ventilation was 2.1 ± 2.9 days (Table 1). Sepsis complicated evolution in 30.7%. The mean length of stay in ICU was 4.9 ± 4.4 days and was almost identical in age groups, with *p*=0.99 (Table 1). Overall mortality was 31.9% (29 cases). Nineteen (65.5%) patients died within 48 hours, of which 12 (41.4%) died within 24 hours. The mean length to death was 3.2 ± 3.5 days. The search for factors associated with death is detailed in Table 3. Demographic factors such as age and sex were not associated with death. On the other hand, the cause of trauma influenced death with 100% (1 case) of deaths for sports accidents, 61.1% for falls, and 23.6% for RTA, with *p*=0.003. Also, secondary hypotensive insult (51.7%), anemia (43.1%), hyperthermia (46.7%), GCS < 6 (64%), and ISS > 20 (48.9%) significantly influenced the death (*p* < 0.05). Finally, the availability of CT scan also impacted death: 56.3% of deaths when CT scan was not available vs. 18.6% when it was (*p*=0.000). The causes of death found are detailed in Table 4. 19 deaths were related only to TBI, when 7 children died of sepsis (mainly acquired ventilation pneumonia) and 3 of hemorrhagic shock. Among the deceased children, 17 had another injury associated with TBI. Among the survivors (Table 5), 39 children were discharged from the hospital without significant neurological sequelae, 18 had behavioral disorders, and 5 remained in a neurovegetative state and were discharged at the parents' request. Worse outcomes globally were related to CT scan nonavailability or initial pGCS below 6 (Table 5).

## 4. Discussion

Severe pTBI in this study accounted for 9.1% of all severe TBI admitted in the multipurpose ICU of SOUH. The pTBI was preferentially affecting the boy, with a mean age of 7.7 years. RTA (79.1%) and falls (19.8%) were the leading causes of pTBI. Transport to hospital was not medicalized, and the pTBI was associated with another injury in 38.5% cases. Death occurred in 31.9%.

A male predominance was found in this study with 67%. El-Menyar et al. found the same male predominance with 4 times more boys than girls. Other studies made the same observations [9–13].

The average age found in this study was 7.7 years, and the most affected age group was that of 5–9 years old with 44%, followed by adolescents aged 10–15 years with 31.8%, when the age group of 0–4 years was the least concerned with 24.2%. Mendy et al. found a similar average age in their study in Dakar (Senegal), including patients aged 0 to 15 years [13]. El-Menyar et al. found an average age of 10 years probably related to the enlargement of their sample to 15–18 years old, with a higher incidence of 40% in the 15- to 18-year age group [9]. The predominance of age groups >5 years in this study, as in those mentioned above, would be related to a higher exposure to RTA, the main cause found of pTBI.

The frequently found causes of pTBI in this study, including RTA with 79% and falls with 20%, are in agreement with those found in most studies [9, 10, 12–14]. These results, however, mask differences between age groups, with the prevalence of RTA above 80% in age groups of >4 years, while falls being the leading cause for 60% in the 0- to 2-year-old group. In El-Menyar et al.'s study in Qatar, all causes related to road traffic accounted for 68.9%, but in children below 4 years, falls predominated with 51.3%. Other French and Asian studies reported the same predominance of falls in children below 2 years and the age group of 3–6 years old [10, 11, 14].

The performed cerebral CT scan rate in this study was 65%. The difficulties of access (not only financial) to this exam not always available in the SOUH could explain this rate. Brain injuries most commonly found when CT was performed were brain edema, skull fracture, and brain contusion, each of which occurred in more than 50% of cases. In El-Menyar et al.'s study, skull fracture, brain contusion, and brain edema were the most common with 62%, 50%, and 31%, respectively. Ducrocq et al. found 32% brain edema and 25% complex injuries in their study [9, 10]. Studies about pTBI reported a high incidence of subarachnoidal hemorrhage and brain contusion, whereas epidural hemorrhage was rare due to a strong dural adhesion to the internal table of the skull [15–17].

The average ISS in this study of 23 was lower than that found by Ducrocq et al. (mean ISS of 28) with 585 pTBI [10]. The reasons lie in the fact that, in our context, in the absence of prehospital care, many severe or critical traumatized children died before arrival at the hospital and these children were not included to this study. The lower rate of associated trauma of 38.5% in this study compared to 52.5% found by Ducrocq et al. supports this observation. The importance of a prehospital system care (which involves the ability to quickly obtain care, rapid and appropriate referrals, and the safe transportation of patients) for a better outcome of injured children is no longer demonstrated. It has been shown today that, wholesale, poorly planned imitations of high-income countries-type prehospital systems in low- and middle-income countries often result in expensive, inefficient systems [18, 19] and, that alternatively, low-cost interventions, for example, first responder training programs in Uganda and Mexico, have resulted in excellent outcomes for relatively low costs [19, 20].

The mortality rate of 34.8% observed in this study is close to that found in Dakar by Mendy et al. While in this study, the observed mortality did not vary by age group, other studies reported differences by age groups with high mortality in youngest age groups [10, 21].

The pTBI mortality rates in resource-limited countries are generally higher or even doubled compared to rich-country rates [3, 22, 23].

Hypotension, anemia, and hyperthermia were the systemic factors of secondary cerebral insults that influenced death in this study. Their role in the occurrence of death has already been demonstrated [10, 24, 25]. Other factors associated with worse outcomes or deaths were pGCS < 6 (64%), ISS > 20 (49%), and the nonavailability of CT scan. Previous studies have reported for high ISS and high mortality; Pigula et al. found 30% of deaths with ISS > 20; Ducrocq et al. found 10% with ISS < 25, 38% for those between 25 and 50, and 83% for that >50. Walker et al. found 44% with ISS > 25 and 0% with that <25. Finally, Waxman et al. found 100% of deaths with ISS > 50 [10, 25–27].

The problem of pTBI in poor countries has been studied and raised a lack of adequate resources for the management of severe trauma leading to high mortality [3, 22]. Solutions must adapt to local conditions so that they are efficient. They go through the establishment of prehospital care systems adapted to our resources, better organization of emergency services and pediatric resuscitation, and training of caregivers to pediatric care, establishing clear protocols to standardize care.

## 5. Conclusion

pTBI mortality remains high in SOUH ICU. Factors associated with mortality were systemic secondary cerebral insults not prevented or not early managed because of the absence of a prehospital care system, worse GCS < 6 and ISS > 20, and the nonavailability of CT scan. In this context, the need for prehospital medicine and efficient emergency services is more than urgent.

## Figures and Tables

**Table 1:** Demographics, mechanism of injury, severity of injury, and complications based on age groups among severe pediatric traumatic brain injury patients.

Variable	Overall	0–4 years	5–9 years	10–15 years	*P* value
Number	91	22 (24.2%)	40 (44%)	29 (31.8%)	
Age (mean ± SD)	7.7 ± 4.3	2.14 ± 0.9	7.0 ± 1.2	12.9 ± 1.8	0.001
Males	61 (67.0%)	13 (59.1%)	27 (67.5%)	21 (72.4%)	0.60
Mechanism of injury					
Road traffic accident	72 (79.1)	13 (59.1%)	35 (87.5%)	24 (82.8%)	0.03
Falls	18 (19.8%)	9 (40.9%)	5 (12.5%)	4 (13.8%)	
Sports accident	1 (1.1%)	0	0	1 (3.4%)	
pGCS (median (range))	7 (3–8)	7 (3–8)	7 (4–8)	7 (4–8)	0.245
ISS (mean ± SD)	23.1 ± 8.4	21.5 ± 7.6	22.6 ± 7.7	24.8 ± 9.6	0.350
Ventilator days (mean ± SD)	2.1 ± 2.9	1.9 ± 1.9	2.4 ± 3.2	2.1 ± 3.2	0.800
ICU stay (mean ± SD)	4.9 ± 4.4	4.8 ± 4.5	5.0 ± 4.0	4.9 ± 5.0	0.99
Sepsis	28 (30.7%)	6 (27.3%)	13 (32.5%)	9 (31.0%)	0.912
Mortality	29 (31.9%)	6 (27.3%)	13 (32.5%)	10 (34.5%)	0.85

SD: standard deviation, pGCS: pediatric Glasgow Coma Scale, ISS: Injury Severity Score, ICU: Intensive Care Unit.

**Table 2:** Type and frequency of head injuries for 59 (64.8%) CT scans done.

Injuries	Overall (%)	Deaths (%)	0–4 years (%)	5–9 years (%)	10–15 years (%)	*P* value
Brain edema	43 (72.9)	8 (18.6)	13 (68.4)	19 (76)	11 (73)	0.85
Skull fracture	41 (69.5)	8 (19.5)	12 (63.2)	19 (76)	10 (66.7)	0.63
Brain contusion	33 (55.9)	4 (12.1)	10 (52.6)	17 (68)	06 (40)	0.21
Subarachnoidal hemorrhage	9 (15.2)	1 (11.1)	4 (21.0)	5 (20)	0	0.163
Intracerebral hemorrhage	8 (13.6)	4 (50)	3 (15.8)	3 (12)	2 (13.3)	0.90
Epidural hemorrhage	4 (6.8)	1 (25)	3 (15.8)	0	1 (6.7)	0.119
Intraventricular hemorrhage	3 (5)	2 (66.7)	0	1 (4)	2 (13.3)	0.203
Diffuse axonal injury	3 (5)	2 (66.7)	2 (10.5)	0	1 (6.7)	0.275
No injury observed	8 (13.6)	0	3 (15.8)	1 (4)	4 (26.7)	0.120

**Table 3:** Factors associated with pediatric traumatic brain injury mortality.

Factors	Deaths (%)	*P* values
Age groups		0.85
0–4 years	6 (27.27)	
5–9 years	13 (32.5)	
10–15 years	10 (34.5)	
Sex		0.324
Male	18 (29.5)	
Female	11 (36.7)	
NIBP (systolic)		0.006
<Normal for age	15 (51.7)	
≥Normal for age	14 (22.6)	
Sa O_2_		0.176
<90%	11 (40.7)	
≥90%	18 (28.1)	
Hemoglobin		0.04
<10 g/dl	19 (43.1)	
≥10 g/dl	10 (22.2)	
Temperature		0.031
≥38°5c	14 (46.7)	
<38°5c	15 (24.6)	
pGCS		0.001
3–5	16 (64)	
6–8	13 (19.7)	
ISS		0.001
≤20	7 (15.2)	
>20	22 (48.9)	
Mechanism of injury		0.003
Road traffic injuries	17 (23.6)	
Falls	11 (61.1)	
Sports accident	1 (100)	
CT scan availability		0.001
Yes	11 (18.6)	
No	18 (56.3)	

NIBP: noninvasive blood pressure; Sa O_2_: peripheral oxygen saturation by using a pulse oximeter; pGCS: pediatric Glasgow Coma Scale; ISS: Injury Severity Score.

**Table 4:** Causes of deaths.

	Number (%)
TBI	19 (65.5)
Non-TBI	10 (34.5)
Sepsis	7 (24.1)
Hemorrhagic shock	3 (10.3)

TBI: traumatic brain injury.

**Table 5:** Outcomes by CT scan availability and initial pediatric Glasgow Coma Scale.

	No significant neurological sequelae (%)	Behavioral disorders (%)	Vegetative state (%)	Death (%)	Total	*P* value
CT scan						0.001
Yes	32 (54.2)	11 (18.6)	5 (8.5)	11 (18.6)	59	
No	7 (21.9)	7 (21.9)	0	18 (56.3)	32	
pGCS						0.001
3–5	3 (12)	4 (16)	2 (8)	16 (64)	25	
6–8	36 (54.5)	14 (21.2)	3 (4.5)	13 (19.7)	66	

CT scan: cerebral computerized tomography; pGCS: pediatric Glasgow Coma Scale.

## Data Availability

The data used to support the findings of this study are available from the corresponding author upon request.

## Abbreviations

pTBI: Pediatric traumatic brain injury
TBI: Traumatic brain injury
SOUH: Sylvanus Olympio University Hospital
ICU: Intensive Care Unit
pGCS: Pediatric Glasgow Coma Scale
ISS: Injury Severity Score
CT scan: Computed tomography scan
RTA: Road traffic accidents.
